# How Did the COVID-19 Lockdown Pandemic Affect the Depression Symptomatology in Mediterranean Older Adults with Metabolic Syndrome?

**DOI:** 10.1155/2023/6765950

**Published:** 2023-07-14

**Authors:** Indira Paz-Graniel, Nancy Babio, Stephanie K. Nishi, Miguel Ángel Martínez-González, Dolores Corella, Montserrat Fitó, Alfredo Martínez, Ángel M. Alonso-Gómez, Julia Wärnberg, Jesús Vioque, Dora Romaguera, José López-Miranda, Ramon Estruch, Francisco J. Tinahones, José Manuel Santos-Lozano, J. Luís Serra-Majem, Aurora Bueno-Cavanillas, Josep A. Tur, Vicente Martín Sánchez, Xavier Pintó, Miguel Delgado-Rodríguez, Pilar Matía-Martín, Josep Vidal, Cristina Calderon-Sanchez, Lidia Daimiel, Emili Ros, Fernando Fernández-Aranda, Estefania Toledo, Cristina Valle-Hita, Jose V. Sorli, Camille Lassale, Antonio Garcia-Rios, Alejandro Oncina-Canovas, Francisco Javier Barón-López, M. Angeles Zulet, Elena Rayó, Rosa Casas, Esther Thomas-Carazo, Lucas Tojal-Sierra, Miguel Damas-Fuentes, Miguel Ruiz-Canela, Sara De las Heras-Delgado, Rebeca Fernandez-Carrión, Olga Castañer, Patricia J. Peña-Orihuela, Sandra Gonzalez-Palacios, Pilar Buil-Cosiales, Albert Goday, Jordi Salas-Salvadó

**Affiliations:** ^1^CIBER de Fisiopatología de la Obesidad y Nutrición, Instituto de Salud Carlos III, Spain; ^2^Universitat Rovira i Virgili, Departament de Bioquímica i Biotecnologia, Unitat de Nutrició, Reus, Spain; ^3^Institut d'Investigació Sanitària Pere Virgili (IISPV), Reus, Spain; ^4^Toronto 3D (Diet, Digestive Tract and Disease) Knowledge Synthesis and Clinical Trials Unit, Toronto, ON, Canada; ^5^Clinical Nutrition and Risk Factor Modification Centre, St. Michael's Hospital, Unity Health Toronto, ON, Canada; ^6^University of Navarra, Department of Preventive Medicine and Public Health, IDISNA, Pamplona, Spain; ^7^Department of Nutrition, Harvard T.H. Chan School of Public Health, Boston, MA, USA; ^8^Department of Preventive Medicine, University of Valencia, Valencia, Spain; ^9^Unit of Cardiovascular Risk and Nutrition, Institut Hospital del Mar de Investigaciones Médicas Municipal d'Investigació Médica (IMIM), Barcelona, Spain; ^10^Department of Nutrition, Food Sciences, and Physiology, Center for Nutrition Research, University of Navarra, Pamplona, Spain; ^11^Navarra Institute for Health Research (IdisNA), 31008 Pamplona, Spain; ^12^Bioaraba Health Research Institute, Cardiovascular, Respiratory and Metabolic Area, Vitoria-Gasteiz, Spain; ^13^Osakidetza Basque Health Service, Araba University Hospital, Vitoria-Gasteiz, Spain; ^14^University of the Basque Country UPV/EHU, Vitoria-Gasteiz, Spain; ^15^EpiPHAAN Research Group, School of Health Sciences, University of Málaga-Instituto de Investigación Biomédica en Málaga (IBIMA), Málaga, Spain; ^16^CIBER de Epidemiología y Salud Pública (CIBERESP), Instituto de Salud Carlos III, Madrid, Spain; ^17^Instituto de Investigación Sanitaria y Biomédica de Alicante, Universidad Miguel Hernandez, ISABIAL-UMH, 03550 Alicante, Spain; ^18^Health Research Institute of the Balearic Islands (IdISBa), Palma de Mallorca, Spain; ^19^Department of Internal Medicine, Maimonides Biomedical Research Institute of Cordoba (IMIBIC), Reina Sofia University Hospital, University of Cordoba, Córdoba, Spain; ^20^Department of Internal Medicine, Institut d'Investigacions Biomèdiques August Pi Sunyer (IDIBAPS), Hospital Clinic, University of Barcelona, Barcelona, Spain; ^21^Institut de Recerca en Nutrició i Seguretat Alimentaria (INSA-UB), University of Barcelona, Barcelona, Spain; ^22^Virgen de la Victoria Hospital, Department of Endocrinology, Instituto de Investigación Biomédica de Málaga (IBIMA), University of Málaga, Málaga, Spain; ^23^Department of Family Medicine, Research Unit, Distrito Sanitario Atención Primaria Sevilla, Sevilla, Spain; ^24^Research Institute of Biomedical and Health Sciences (IUIBS), University of Las Palmas de Gran Canaria & Centro Hospitalario Universitario Insular Materno Infantil (CHUIMI), Canarian Health Service, Las Palmas de Gran Canaria, Spain; ^25^Department of Preventive Medicine and Public Health, University of Granada, Granada, Spain; ^26^Research Group on Community Nutrition & Oxidative Stress, University of Balearic Islands, Palma de Mallorca, Spain; ^27^Institute of Biomedicine (IBIOMED), University of León, León, Spain; ^28^Lipids and Vascular Risk Unit, Internal Medicine, Hospital Universitario de Bellvitge-IDIBELL, Hospitalet de Llobregat, Barcelona, Spain; ^29^Department of Clinical Sciences, School of Medicine and Health Sciences, University of Barcelona, Hospitalet de Llobregat, 08907 Barcelona, Spain; ^30^Division of Preventive Medicine, Faculty of Medicine; Instituto Universitario de Investigación en Olivar y Aceites de Oliva (INUO), University of Jaén, Jaén, Spain; ^31^Department of Endocrinology and Nutrition, Instituto de Investigación Sanitaria Hospital Clínico San Carlos (IdISSC), Universidad Complutense, Madrid, Spain; ^32^CIBER Diabetes y Enfermedades Metabólicas (CIBERDEM), Instituto de Salud Carlos III (ISCIII), Madrid, Spain; ^33^Department of Endocrinology, Institut d'Investigacions Biomédiques August Pi Sunyer (IDIBAPS), Hospital Clinic, University of Barcelona, Barcelona, Spain; ^34^Department of Endocrinology and Nutrition, Hospital Fundación Jimenez Díaz, Instituto de Investigaciones Biomédicas IISFJD, University Autonoma, Madrid, Spain; ^35^Nutritional Control of the Epigenome Group, Precision Nutrition and Obesity Program, IMDEA Food, CEI UAM + CSIC, Madrid, Spain; ^36^Lipid Clinic, Department of Endocrinology and Nutrition, Institut d'Investigacions Biomèdiques August Pi Sunyer (IDIBAPS), Hospital Clínic, Barcelona, Spain; ^37^Psychoneurobiology of Eating and Addictive Behaviors Group, Institut d'Investigació Biomèdica de Bellvitge (IDIBELL), 08907 Barcelona, Spain; ^38^Clinical Psychology Unit, University Hospital of Bellvitge, 08907 Barcelona, Spain; ^39^Department of Medicine and Life Sciences, Universitat Pompeu Fabra (UPF), 08003 Barcelona, Spain; ^40^Centro de Salud Gran Capitán, Servicio Andaluz de Salud, Spain; ^41^Atención Primaria, Servicio Navarro de Salud, Spain

## Abstract

**Background and Aims:**

To control the COVID-19 spread, in March 2020, a forced home lockdown was established in Spain. In the present study, we aimed to assess the effect of mobility and social COVID-19-established restrictions on depressive symptomatology in older adults with metabolic syndrome. We hypothesize that severe restrictions might have resulted in detrimental changes in depressive symptomatology.

**Methods:**

2,312 PREDIMED-Plus study participants (men = 53.9%; mean age = 64.9 ± 4.8 years) who completed a COVID-19 lockdown questionnaire to assess the severity of restrictions/lockdown and the validated Spanish version of the Beck Depression Inventory-II (BDI-II) during the three established phases concerning the COVID-19 lockdown in Spain (prelockdown, lockdown, and postlockdown) were included in this longitudinal analysis. Participants were categorized according to high or low lockdown severity. Analyses of covariance were performed to assess changes in depressive symptomatology across lockdown phases.

**Results:**

No significant differences in participant depression symptomatology changes were observed between lockdown severity categories (low/high) at the studied phases. During the lockdown phase, participants showed a decrease in BDI-II score compared to the prelockdown phase (mean (95% CI), -0.48 (-0.24, -0.72), *P* < 0.001); a nonsignificantly larger decrease was observed in participants allocated in the low-lockdown category (low: -0.59 (-0.95, -0.23), high: -0.43 (-0.67, -0.19)). Similar decreases in depression symptomatology were found for the physical environment dimension. The post- and prelockdown phase BDI-II scores were roughly similar.

**Conclusions:**

The COVID-19 pandemic lockdown was associated with a decrease in depressive symptomatology that returned to prelockdown levels after the lockdown. The degree of lockdown was not associated with depressive symptomatology. The potential preventive role of the physical environment and social interactions on mental disorders during forced home lockdown should be further studied. This trial is registered with ISRCTN89898870. Retrospectively registered on 24 July 2014.

## 1. Introduction

The global outbreak of the coronavirus disease 2019 (COVID-19) was declared by the World Health Organization early in 2020 [1]. To control COVID-19's spread, worldwide lockdowns and curfews were established [2]. In Spain, a shelter-in-place order was enforced from March 14th to June 21st, 2020, followed by a transition plan to shift from severe lockdown to less restrictive conditions in accordance with COVID-19 prevalence/incidence and pressure on the healthcare system [2, 3]; during 2021, the severity of restrictive conditions varied according to regional sanitary conditions [4]. The applied social and mobility restrictions, in addition to the fear of contagion, were prone to promoting population mental distress. In this line, the risk of developing depression has been associated with adverse life conditions and events (poverty, loneliness, unemployment, loss of a loved one, illness, alcohol use, etc.) [5]. Therefore, the pandemic was the ideal scenario for depression onset. It was reported that during the COVID-19 pandemic, the global prevalence of depression disorder increased by an estimated 27.6% [6]. Special concerns regarding older adults' mental health were raised as it was suggested that this population might be more susceptible to greater adverse effects from COVID-19 [7]. However, controversial results on depression status in elderly populations during the first months of the pandemic had been reported [8] [9]. It should be noted that most of these results are cross-sectional, reflecting the acute impact of COVID-19 on depression symptomatology without comparisons with scores before the COVID-19 pandemic and driven from self-reported data collected through online questionnaires [9]. To the best of our knowledge, only one study has compared depression prevalence in the general Spanish population before and after the pandemic started [10]. According to this report, the population reporting depressive symptoms increased from 5.75% (December 2019) to 8.84% (December 2020) [10]; however, the impact of the social and mobility restrictions on depression was not explored. In 2020, Ding et al. [11] conducted a cross-sectional multicountry study to explore the relationship between government restrictions during the COVID-19 pandemic and depression symptomology, but no association was observed.

Because of the limited evidence on the long-term COVID-19 lockdown impact on depressive symptomatology in vulnerable populations such as older adults, the study of the impact of the social and mobility restrictions on depression might be helpful in understanding the mental health effects of the pandemic on this population. In this report, we aimed to describe in older Spanish adults the changes in depression symptomatology during the COVID-19 pandemic according to categories of lockdown strictness. We hypothesized that individuals who experienced a severe lockdown and social restrictions would present higher detrimental changes in depressive symptomatology in comparison with those who were exposed to less severe lockdown conditions.

## 2. Methods

### 2.1. Study Design and Participants

The present study was conducted within the framework of the PREDIMED-Plus study. Briefly, the PREDIMED-Plus is an ongoing multicenter, randomized, controlled intervention trial conducted in Spain, which aims to assess the effect of an intensive lifestyle intervention on the primary prevention of CVD in comparison to advice on the Mediterranean diet. From October 2013 to October 2016, 6874 participants were recruited and randomized in 23 centers from various universities, hospitals, and research institutes in Spain. The eligible participants were women and men (age 55–75 years old) with overweight/obesity (body mass index (BMI) 27–40 kg/m^2^) and metabolic syndrome (MetS) [12] [13] and were free of cardiovascular disease (CVD) at baseline. The trial design and inclusion and exclusion criteria have been extensively detailed [14].

The present analysis was performed with data from participants who underwent the follow-up visits encompassed the three identified phases concerning the COVID-19 pandemic situation in Spain: prelockdown (March-December 2019, no sanitary restrictions), lockdown (March-December 2020, the most severe sanitary restrictions), and postlockdown (March-December 2021, less severe restrictions). In December 2021, the only sanitary policy maintained was the mandatory use of a facemask [15]. Participants without scheduled follow-up visits in any of the aforementioned phases, those who did not complete the Beck Depression Inventory-II (BDI-II), or those who did not answer the PREDIMED-Plus COVID-19 lockdown questionnaire were excluded from the analyses (*n* = 4,562) (Supplemental Figure 1).

### 2.2. Exposure: Lockdown Severity

The main independent variable was lockdown severity, based on a lockdown score. Between June and August 2020, a questionnaire aiming to collect data on the conditions which study participants experienced during lockdown was completed by enrolled participants who attended in-person visits. Five items from this questionnaire were used to create the lockdown score: (a) household size (m^2^, cut-off points ≥ 95 m^2^, <95 m^2^, score from 0 to 1), (b) housing conditions (to have windows, balcony, terrace, and/or garden, score from 0 to 4), (c) frequency in which participants left home during lockdown (“more than once a day,” “<1 time a day,” “twice in 15 days,” and “never,” score from 0 to 3), (d) the number of people cohabiting with the participant during lockdown (“>2,” “1-2,” and “0,” score from 0 to 2), and (e) employment status during the lockdown (“currently working,” “part-time working,” and “retired,” score from 0 to 2). The first 3 items comprised the physical-environment dimension, while the last two comprised the social-contact dimension. The overall lockdown score was constructed using the sum of the 5 items; the higher the score, the more severe the lockdown (Supplemental Table 1). Finally, based on the distribution of the variables, the overall lockdown score was divided into high (11 to ≥7) or low (6 to 1), the physical environment score into high (7 to ≥5) or low (4 to 0), and the social contact score into high (4 to ≥3) or low (3 to 0).

### 2.3. Outcome: Depressive Symptomatology

Changes in depressive symptomatology were the outcome of interest. Depressive symptomatology was assessed yearly using the validated Spanish version of the BDI-II [16]. This score includes 21 questions with four possible answers sorted according to the severity of symptoms; the score ranges from 0 to 63 points, with higher values indicating greater depression symptomatology. To assess changes in depressive symptomatology, the differences in BDI-II punctuation among lockdown phases (lockdown and postlockdown vs. prelockdown) were estimated. The threshold for moderate-severe depression status was set at BDI-II score ≥ 19 [16].

### 2.4. Covariates Assessment

At baseline and yearly, all participants provided information on sociodemographic characteristics, lifestyle, physical activity (PA) [17], health status, and adherence to an energy reduced Mediterranean diet score (17-item MedDiet score) [18], among others. Anthropometric data was collected according to the study protocol, and BMI was calculated by dividing weight (kg) by height squared (m^2^).

### 2.5. Statistical Analysis

For the present study, the PREDIMED-Plus database updated until December 2021 was used. To compare the baseline characteristics of the study participants in terms of lockdown strictness, the *x*^2^ test and Student's *t*-test were used, as appropriate. The differences in changes in depressive symptomatology were evaluated by analysis of covariance (ANCOVA) adjusted by sex, age, intervention group, previous BDI-II score, center, civil status, educational level, smoking status, previous self-reported depression diagnosis, and change in MedDiet adherence. The data were analysed using the Stata 14 software (StataCorp, College Station, TX, USA), and statistical significance was set at a two-tailed *P* value <0.05.

### 2.6. Ethics

All participants provided their written informed consent. The study protocol and procedures were approved by the institutional review boards of each participating center in accordance with the ethical standards of the Declaration of Helsinki. The studies involving human participants were reviewed and approved by the CEI Provincial de Málaga-Servicio Andaluz de Salud O01_feb_PR2 - Predimedplus nodo 1 CEI de los Hospitales Universitarios Virgen Macarena y Virgen del Rocío-Servicio Andaluz de Salud PI13/00673 CEIC Universidad de Navarra 053/2013 CEI de las Illes Balears - Conselleria de Salut Direcció General de Salut Publica i Consum IB 2242/14 PI CEIC del Hospital Clínic de Barcelona HCB/2016/0287 CEIC Parc de Salut Mar y IDIAP Jordi Gol PI13/120 CEIC del Hospital Universitari Sant Joan de Reus y IDIAB Jordi Gol 13-07-25/7proj2 CEI de la Provincia de Granada- Servicio Andaluz de Salud MAB/BGP/pg CEIC de la Fundacion Jiménez Díaz EC 26-14/IIS-FJD CEIC Universidad de Navarra 053/2013 CEIC Euskadi PI2014044 CEIC Corporativo de Atención Primaria de la Comunitat Valenciana 2011-005398-22 CEI Humana de la Universidad de las Palmas de Gran Canaria CEIH-2013-07 CEIC del Hospital de Bellvitge PR240/13 CEI de Cordoba-Junta de Salud 3078 CEI de la Fundación IMDEA Alimentación PI-012 CEIC Hospital Clínico San Carlos de Madrid-Piloto-CEIC Servicio Madrileño de salud-General 30/15 CEI Provincial de Málaga-Servicio Andaluz de Salud CEI de las Illes Balears - Conselleria de Salut Direcció General de Salut Publica i Consum IB 2251/14 PI CEIC del Hospital Clínic de Barcelona HCB/2017/0351 CEIC del Hospital General Universitario de Alicante CEIC PI2017/02 CEIC de la Investigación Biomédica de Andalucía (CCEIBA) CEI de la Universidad de León ÉTICA-ULE-014-2015. The participants provided their written informed consent to participate in this study.

## 3. Results

A total of 2,312 participants (men, 53.9%; mean age, 64.9 ± 4.8 years) were included in the present analysis. The general and lifestyle characteristics of the studied population before the lockdown according to categories of lockdown are presented in Table 1. 68.2% of the studied population was categorized into the high-lockdown category. Significant differences were observed among exposure categories, except for BMI and adherence to the MedDiet. Participants in the high-lockdown category were older, more likely to be women, and showed higher scores on the BDI-II compared to participants allocated to the low-severity lockdown category.

Supplemental Table 2 displays the mean and SD BDI-II score across lockdown phases. A total of 55 participants increased their BDI-II score ≥ 19 during the lockdown phase, from which about 60% returned to normal levels during the postlockdown phase.

**Figure 1:**
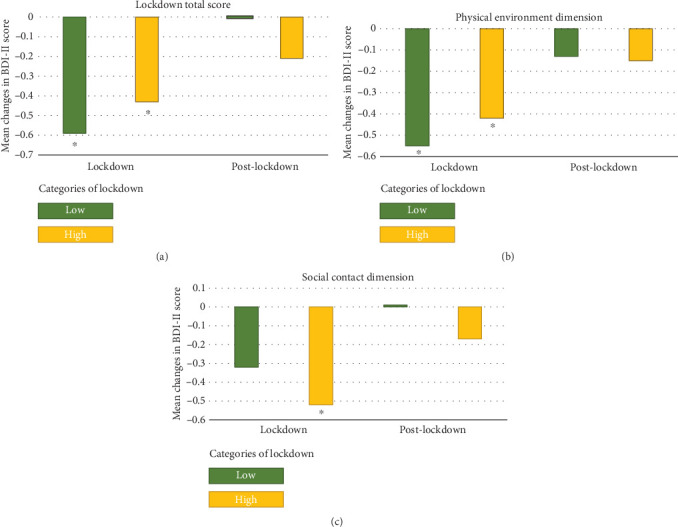
Mean changes in BDI-II score in Spanish older adults by studied phases (lockdown and postlockdown) and according to categories (low/high) of total lockdown score, physical environment, and social contact dimensions. ANCOVA adjusted for sex, age, intervention group, previous depression symptomatology, civil status, educational level, smoking habit, previous depression diagnosis, changes in MedDiet adherence, and recruitment center. ⁣^∗^Significant changes in comparison to the prelockdown BDI-II score. Lockdown represents changes between lockdown vs. prelockdown BDI-II scores; postlockdown represents changes between postlockdown vs. prelockdown BDI-II scores.

Figure 1 and Supplemental Table 3 display the mean participant changes in BDI-II score by studied phases and according to categories of total lockdown, physical environment, and social contact dimensions. No significant differences in changes in depression symptomatology between participants in lockdown categories (low/high) at the studied phases (prelockdown, lockdown, and postlockdown) were shown. During the lockdown phase, participants showed a significant decrease in BDI-II score compared to the prelockdown phase (mean (95% CI), -0.48 (-0.24, -0.72), *P* < 0.001); however, a nonsignificant larger decrease was observed in participants allocated in the low-lockdown category (low: -0.59 (-0.95, -0.23), high: -0.43 (-0.67, -0.19)).

Similar changes in the BDI-II score were observed for the physical-environment dimension during the lockdown compared to the prelockdown phase (mean (95% CI), low: -0.50 (-0.81, -0.27), high: -0.42 (-0.71, -0.13)). For the social dimension, compared to the prelockdown phase, during the lockdown, a significant decrease (-0.52 (-0.73, -0.30)) in BDI-II score was observed in participants allocated to the high-severity lockdown category but not in those allocated to the low-severity category (-0.32 (-0.81, 0.17)). When the participant's postlockdown BDI-II score was compared to the prelockdown BDI-II score, no significant changes were observed across the categories of total lockdown or in any of the explored dimensions.

## 4. Discussion

In this study conducted on a large sample of older Spanish adults with MetS, we observed that the BDI-II score decreased during the lockdown phase in comparison to the prelockdown phase, and a nonsignificantly larger decrease was observed for individuals allocated to the “low-severity lockdown” category. During the postlockdown phase, the participant's BDI-II returned to prelockdown levels, with no significant changes observed between categories of total lockdown nor in none of the physical or social dimensions. Importantly, the mean BDI-II scores across all 3 phases remained in the same relative category (no to minimal depression symptoms experienced).

It has been reported that depression prevalence increases with aging (women > 7.5% vs. >5.5% men) [19]. Therefore, it was thought that the pandemic lockdown might promote the onset/worsening of mental disorders in the aged population, as these individuals were also identified to be at increased risk for COVID-19 complications [20]. In this regard, findings from a pooled analysis including data from 7 cohorts (*n* = 7,419) showed that standardised mean difference change (mean (95% CI)) for depression symptomatology in older adults before and during the COVID-19 pandemic was 0.22 (0.06; 0.38), *I*^2^ = 95%) [9]. Nevertheless, this was not observed in our population; on the contrary, a decrease in depression symptomatology was shown during the hardest lockdown phase (March-December 2020). Similar to our results, in a cross-sectional study conducted on older Dutch individuals, it was reported that mental health status remained stable during the COVID-19 lockdown (before vs. after the pandemic), although an increased loneliness perception was reported [21]. Furthermore, we did not detect a significant difference in the BDI-II among lockdown severity categories, which is in line with results from a previous multicountry survey conducted by Ding et al. [11]. In that report, no associations were observed between governmental restrictions applied during the COVID-19 pandemic and depressive symptoms [11]. However, results from a cross-sectional analysis conducted in a German population (*n* = 4335, women = 75.8%, mean age = 40.5 years) during the first COVID-19 wave showed that a stronger reduction of social contacts, higher distress due to restrictions on social contacts, stronger perceived changes in life due to the public health measures, and a more negative appraisal of these perceived changes were positively associated with higher depressive symptomatology [22]. Discrepancies noticed between our study and other studies might be due to differences in the lookdown severity, methodology used, the study design, and sample characteristics.

Potential explanations have been proposed for these observations. First, an older population might have been less likely to be affected by the social and economic consequences of the pandemic than younger individuals [23]. In general, lower rates of depression prevalence during the first months of the pandemic were reported for older adults in comparison with younger adults in Europe and the USA [23, 24]. Second, older adults with known depression or anxiety were shown to be more resilient to the mental health effects of COVID-19 thanks to previous knowledge of coping strategies, social connectedness, and mental healthcare [25]. In addition, older adults with higher BDI-II scores or at increased risk of COVID-19 infection complications might have found it a relief to stay at home and not carry out regular tasks and social interactions. Furthermore, during the lockdown, our study participants were often contacted by trial staff to receive advice on adherence to a healthy lifestyle (promotion of MedDiet, home training, etc.). The adherence and maintenance of healthy habits might have played an important role in depression prevention, as has been reported previously [26]. Moreover, it has been proposed that the augment in the frequency and quality of the relationships (including remote interactions) which occurred during the lockdown might have prevented depression onset [21]. Nonetheless, these interactions decreased during the postlockdown. Our results regarding the social-contact dimension might be a reflection of this.

Our study has certain limitations that must be considered. Due to the sample characteristics and the PREDIMED-Plus design, results cannot be generalized. Second, despite the BDI-II score being one of the most commonly used assessment tools regarding depression status in epidemiological research, it cannot substitute for a complete clinical diagnosis. Third, women participants were underrepresented in the low-severity lockdown category, which might have induced some bias. Fourth, participants who were not able to attend follow-up in-person visits may have had more severe restrictions and/or greater depressive symptoms which were not possible to assess. Finally, we cannot discard that the observed results might be affected by residual confounding. Despite these limitations, it should be highlighted that, to our knowledge, this is the first large epidemiological study to report changes in depression symptomatology in adults using prospective data collected in face-to-face interviews through a validated tool.

## 5. Conclusions

In summary, in a Spanish older population with MetS, the COVID-19 pandemic lockdown was associated with a modest decrease in the BDI-II score that returned to prelockdown levels after the lockdown. The degree of lockdown was not associated with depressive symptomatology. Further studies are needed to clarify the mechanism beyond the potential role of the physical environment and social interactions on mental disorders during the forced home lockdown.

## Figures and Tables

**Table 1:** General and lifestyle characteristics of the studied population according to categories of lockdown.

	Lockdown score (11 to 1 points)			
	Full sample *n* = 2,312	Low (6 to 1) *n* = 737	High (11 to ≥7) *n* = 1,575	*P* value
Age at baseline, years	64.9 ± 4.8	63.3 ± 4.9	65.6 ± 4.6	<0.01
Men, % (*n*)	1,246 (53.9%)	72.6 (535)	45.1 (711)	<0.01
BDI-II score				
Pre COVID-19 lockdown	6.8 ± 7.1	5.8 ± 6.6	7.2 ± 7.3	<0.01
During COVID-19 lockdown	6.3 ± 6.2	5.4 ± 5.7	6.7 ± 6.3	<0.01
Post COVID-19 lockdown	6.6 ± 6.5	5.9 ± 6.1	7.0 ± 6.6	<0.01
BDI-II ≥19^b^, % (*n*)				
Pre COVID-19 lockdown	5.9 (137)	4.8 (35)	6.5 (102)	0.10
During COVID-19 lockdown	4.5 (105)	3.3 (24)	5.1 (81)	0.04
Post COVID-19 lockdown	4.7 (109)	3.8 (28)	5.1 (81)	0.15
History of depression, % (*n*)	21.6 (500)	17.6 (130)	23.5 (370)	<0.01
BMI^t^, kg/m^2^	31.7 ± 3.8	31.7 ± 4.0	31.9 ± 3.8	0.57
Education level, % (*n*)				<0.01
Less than high school	48.7 (1,125)	39.6 (292)	52.9 (833)	
High school or equivalent	29.5 (682)	30.9 (228)	28.8 (454)	
University	21.8 (505)	29.4 (217)	18.3 (288)	
Smoking habit, % (*n*)				<0.01
Never smoker	44.4 (1,026)	33.9 (250)	49.3 (776)	
Former smoker	43.5 (1,005)	49.7 (366)	40.6 (639)	
Current smoker	12.2 (281)	16.4 (121)	10.2 (160)	
Civil status, % (*n*)				<0.01
Single or divorced	10.8 (249)	8.7 (64)	11.8 (185)	
Married	80.1 (1,848)	86.0 (633)	77.3 (1215)	
Widower	9.1 (210)	5.3 (39)	10.9 (171)	
Leisure-time physical activity^t^, MetS. min./day	458.5 ± 369.2	500.6 ± 411.8	432.2 ± 367	<0.01
Physical activity^t^, min./day				
High intensity^t^, MetS. min./week	153.2 ± 239.9	159.5 ± 242.6	150.2 ± 238.7	0.38
Moderate intensity^t^, MetS. min./week	171.7 ± 246.5	194.2 ± 272.3	161.2 ± 232.8	<0.01
Low intensity^t^, MetS. min./week	133.6 ± 147.3	135.8 ± 163.1	132.6 ± 139.3	0.62
17-item MedDiet score^t^	11.9 ± 2.8	11.9 ± 2.9	12.0 ± 2.7	0.29
Alcohol consumption^t^, g ethanol/d	10.0 ± 13.9	12.0 ± 14.2	9.0 ± 13.6	<0.01
Intervention group	49.2 (1,137)	48.9 (360)	49.3 (777)	0.04

BDI-II score: Beck Depression Inventory-II; BMI: body mass index; COVID-19: coronavirus disease 2019; MedDiet: Mediterranean diet. ^a^*P* values for comparisons between groups were tested by Student's *t*-test or *χ*^2^ as appropriate. ^b^The participants is considered to suffer depression when getting a BDI-II score of 19 or higher. ^t^Data during the prelockdown phase. There were missing data for civil status in 5 (0.22%), BMI in 1 (0.04%), physical activity in 2 (0.08%), adherence to MedDiet in 4 (0.17%), and alcohol consumption in 358 (15.5%) participants.

## Data Availability

The datasets generated and analyzed during the current study are not expected to be made available outside the core research group, as neither participants' consent forms nor ethics approval included permission for open access. However, the researchers will follow a controlled data-sharing collaboration model, as in the informed consent participants agreed with a controlled collaboration with other investigators for research related to the project's aims. Therefore, investigators who are interested in this study can contact the PREDIMED-Plus steering committee by sending a request letter to predimed_plus_scommittee@googlegroups.com. A data-sharing agreement indicating the characteristics of the collaboration and data management will be completed for the proposals that are approved by the steering committee.

## Supplementary Materials

Supplementary Materials Supplemental Figure 1: flow diagram for study participants. Abbreviations: BDI-II score: Beck Depression Inventory-II; P-Plus: PREDIMED-Plus study. ^Ɨ^Studied phases: prelockdown (from March to December 2019), lockdown (from March to December 2020), and postlockdown (from March to December 2021). Supplemental Table 1: creation of the lockdown score. Abbreviations: P-Plus: PREDIMED-Plus. Supplemental Table 2: mean BDI-II score across confinement phases (*n* = 2,312). Abbreviations: BDI-II score: Beck Depression Inventory-II; COV: lockdown phase; POST: postlockdown phase; PRE: prelockdown phase. *t* values are means ± SDs. ^a^Data analyzed by 2-factor repeated-measures ANOVA (*P* < 0.05). ^b^Between-group difference in change by the Bonferroni post hoc test (*P* < 0.05). Supplemental Table 3: mean changes in BDI-II score in Spanish older adults according to categories of total lockdown, social contact, and physical environment dimensions during the COVID-19 pandemic. Abbreviations: COV: lockdown phase; PRE: prelockdown phase; POST: postlockdown phase. ANCOVA adjusted for sex (men or women), age at baseline (years), intervention group (control/intervention), previous depression symptomatology (BDI-II score), civil status (single, married, or widower), educational level (less than high school, high school, or university), smoking habit (never, former, or current smoker), previous depression diagnosis (yes/no), changes in MedDiet adherence (category of change), and recruitment center (in quartiles by the number of participants). ^Ɨ^*P* value for comparison between low vs. high lockdown categories. ⁣^∗^Significant changes in comparison to prelockdown BDI-II score (*Pvalue* < 0.05). ^a^In the analyses, there were missing data for civil status in 5 participants (0.22%) and adherence to MedDiet in 6 participants (0.26%). ^b^In the analyses, there were missing data for civil status in 5 participants (0.22%) and adherence to MedDiet in 9 participants (0.39%). Differences in changes between groups analyzed by repeated-measures ANOVA: time × total lockdown categories, *P* value = 0.59; time × social contact dimension, *P* value = 0.65; and time × physical environment dimension, *P* value = 0.32.
