# Supplementary material for: How Did the COVID-19 Lockdown Pandemic Affect the Depression Symptomatology in Mediterranean Older Adults with Metabolic Syndrome?
Source: Depress Anxiety. 2023 Jul 14;2023:6765950. doi: 10.1155/2023/6765950 (PMC11921839; doi:10.1155/2023/6765950)
Supplement: Supplementary Materials — Supplemental Figure 1: flow diagram for study participants. Abbreviations: BDI-II score: Beck Depression Inventory-II; P-Plus: PREDIMED-Plus study. ƗStudied phases: prelockdown (from March to December 2019), lockdown (from March to December 2020), and postlockdown (from March to December 2021). Supplemental Table 1: creation of the lockdown score. Abbreviations: P-Plus: PREDIMED-Plus. Supplemental Table 2: mean BDI-II score across confinement phases (n = 2,312). Abbreviations: BDI-II score: Beck Depression Inventory-II; COV: lockdown phase; POST: postlockdown phase; PRE: prelockdown phase. t values are means ± SDs. aData analyzed by 2-factor repeated-measures ANOVA (P < 0.05). bBetween-group difference in change by the Bonferroni post hoc test (P < 0.05). Supplemental Table 3: mean changes in BDI-II score in Spanish older adults according to categories of total lockdown, social contact, and physical environment dimensions during the COVID-19 pandemic. Abbreviations: COV: lockdown phase; PRE: prelockdown phase; POST: postlockdown phase. ANCOVA adjusted for sex (men or women), age at baseline (years), intervention group (control/intervention), previous depression symptomatology (BDI-II score), civil status (single, married, or widower), educational level (less than high school, high school, or university), smoking habit (never, former, or current smoker), previous depression diagnosis (yes/no), changes in MedDiet adherence (category of change), and recruitment center (in quartiles by the number of participants). ƗP value for comparison between low vs. high lockdown categories. ⁣∗Significant changes in comparison to prelockdown BDI-II score (Pvalue < 0.05). aIn the analyses, there were missing data for civil status in 5 participants (0.22%) and adherence to MedDiet in 6 participants (0.26%). bIn the analyses, there were missing data for civil status in 5 participants (0.22%) and adherence to MedDiet in 9 participants (0.39%). Differences in changes between groups analy [file 6765950.f1.docx]

**Supplemental Material**

- **Supplemental Figure 1.** Flow diagram for study participants.
- **Supplemental Table 1.** Creation of the lockdown score
- **Supplemental Table 2.** Mean BDI-II score across confinement phases (n= 2,312)
- **Supplemental Table 3.** Mean changes in BDI-II score in Spanish older adults according to categories of total lockdown, social contact and physical environment dimensions during the COVID19 pandemic.

**Supplemental Figure 1. Flow diagram for study participants.**

**6,874** participants enrolled in the P-Plus study

Participants without follow-up visit on any studied phase^ɬ^ n = 2,262

**4,612** participants with data at the 3 studied phases

Participants without data on BDI-II score at any study phase^ɬ^, n = 1,577

**3,035** participants with data on BDI-II score at the 3 studied phases

Participants who did not complete the COVID-19 lockdown questionnaire, n = 723

Final sample n = **2,312**

Abbreviations: BDI-II score, Beck Depression Inventory-II; P-Plus, PREDIMED-Plus study.

^Ɨ^ Studied phases: Pre- lockdown (from March to December 2019), lockdown (from March to December 2020) and post- lockdown (from March to December 2021).

| **Supplemental Table 1.** Creation of the lockdown score | | | | | |
| --- | --- | --- | --- | --- | --- |
| Maximum index points | Physical environment dimension | | | Social contact dimension | |
|  | Household size | Housing conditions | Going out frequency | No. people co-habiting with P-Plus participant | Employment status |
| 4 | - | None | - | - | - |
| 3 | - | To have windows | Never | - | - |
| 2 | - | Windows + balcony | Twice in 15 days | 0 | Retired-job loss-unemployment benefits |
| 1 | <95 m^2^ | Windows, balcony + terrace | <1 time a day | 1-2 | Part-time working |
| 0 | ≥95 m^2^ | Windows, balcony, terrace + garden | > once a day | >2 | Currently working |
| Abbreviations: P-Plus, PREDIMED-Plus | | | | | |

| **Supplemental Table 2.** Mean BDI-II score across confinement phases (n = 2,312) | | | | | | | |
| --- | --- | --- | --- | --- | --- | --- | --- |
|  | Pre-lockdown | Lockdown | Post- lockdown | *P-value*^a^ | *COV vs. PRE*^b^ | *POST vs. PRE*^b^ | *POST vs. COV*^b^ |
| **BDI-II score**^t^ | 6.8 ± 7.1 | 6.3 ± 6.2 | 6.6 ± 6.5 | 0.03 | 0.04 | 1.00 | 0.217 |
| Abbreviations: BDI-II score, Beck Depression Inventory II; COV, lockdown phase; POST, post-lockdown phase; PRE, pre-lockdown phase. ^t^ Values are means ± SDs.  ^a^Data analyzed by 2-factor repeated-measures ANOVA (*P*<0.05)  ^b^Between-group difference in change by the Bonferroni post hoc test (*P*<0.05) | | | | | | | |

| **Supplemental Table 3.** Mean changes in BDI-II score in Spanish older adults according to categories of total lockdown, social contact and physical environment dimensions during the COVID19 pandemic. | | | | | | | | | | | |
| --- | --- | --- | --- | --- | --- | --- | --- | --- | --- | --- | --- |
|  | | Lockdown score | | | Social contact | | | Physical environment | | | |
|  | Low (<7) | | High (≥7) | P-value^Ɨ^ | Low (<3) | High (≥3) | P-value^Ɨ^ | | Low (<5) | High (≥5) | P-value^Ɨ^ |
| Participants, n | 737 | | 1575 |  | 387 | 1925 |  | | 1225 | 1087 |  |
| COV vs. PRE  (n = 2,301) ^a^ | **-0.59 (-0.95, -0.23)*** | | **-0.43 (-0.67, -0.19)*** | 0.48 | -0.32 (-0.81, 0.17) | **-0.52 (-0.73, -0.30)*** | 0.48 | | **-0.50 (-0.81, -0.27)*** | **-0.42 (-0.71, -0.13)*** | 0.54 |
| POST vs. PRE  (n = 2,298) ^b^ | 0.01 (-0.37, 0.39) | | -0.21 (-0.46, 0.05) | 0.35 | 0.01 (-0.52, 0.53) | -0.17 (-0.39, 0.06) | 0.55 | | -0.13 (-0.42, 0.16) | -0.15 (-0.46, 0.16) | 0.92 |
| Abbreviations: COV, lockdown phase; PRE, pre-lockdown phase; POST, post- lockdown phase. ANCOVA adjusted for sex (men or women), age at baseline (years), intervention group (control/intervention), previous depression symptomatology (BDI-II score), civil status (single, married, or widower), educational level (less than high school, high school, or university), smoking habit (never, former or current smoker), previous depression diagnosis (yes/no), changes in MedDiet adherence (category of change) and recruitment center (in quartiles by number of participants). ^Ɨ^ *P-value* for comparison between low vs. high lockdown categories. ^*^ Significant changes in comparison to pre- lockdown BDI-II score (*P-value* < 0.05).  ^a^ In the analyses, there were missing data for civil status in 5 participants (0.22%), and adherence to MedDiet in 6 participants (0.26%).  ^b^ In the analyses, there were missing data for civil status in 5 participants (0.22%), and adherence to MedDiet in 9 participants (0.39%).  Differences in changes between group analyzed by repeated-measures ANOVA: Time x total lockdown categories, *P-value* = *0.59;* Time x social contact dimension, *P-value* = *0.65;* and Time x physical environment dimension, *P-value* = *0.32*. | | | | | | | | | | | |
